# Case Report: Beckwith–Wiedemann syndrome with reduced *H19* expression

**DOI:** 10.3389/fped.2025.1690760

**Published:** 2025-11-04

**Authors:** Meng Wang, Jiegang Deng, Shuhua Xing, Xuening Niu

**Affiliations:** Department of Cardiology, Tianjin Children’s Hospital (Tianjin University Children’s Hospital), Tianjin, China

**Keywords:** Beckwith–Wiedemann syndrome, umbilical hernia, atrial tachycardia, imprinting control region 1 hypermethylation, tumor surveillance

## Abstract

**Background:**

Beckwith–Wiedemann syndrome (BWS) is a congenital imprinting disorder characterized by macrosomia, umbilical hernia, macroglossia, and increased tumor susceptibility. DNA methylation changes at 11p15.5 are its primary molecular mechanisms. This report presents a case of BWS wherein the patient registered a gain of methylation at imprinting control region 1 (IC1) and reduced *H19* expression, along with typical clinical features and recurrent atrial tachycardia.

**Case presentation:**

A 2-month-old female infant presented with macroglossia, umbilical hernia, organomegaly, and arrhythmia. She had a history of fetal macrosomia and polyhydramnios. Echocardiography revealed a patent ductus arteriosus, and an electrocardiogram confirmed atrial tachycardia. Multiplex ligation–dependent probe amplification testing showed normal copy number at 11p15.5 but a gain of methylation at IC1, confirming the diagnosis of BWS. When the patient was 12 months old, a tongue reduction surgery with ablation was performed because of feeding and speech difficulties experienced by the patient. A follow-up examination at 18–20 months showed no evidence of tumor development, improved pronunciation, and recurrence of atrial tachycardia without myocardial hypertrophy.

**Conclusion:**

This case underscores the diagnostic value of methylation testing in BWS, especially when the copy number is normal. Infants with macrosomia, macroglossia, and umbilical hernia should be evaluated for BWS. Long-term multidisciplinary follow-up, including tumor surveillance and cardiac monitoring, is essential for improving prognosis and quality of life.

## Introduction

Beckwith–Wiedemann syndrome (BWS) is a congenital imprinting disorder caused by epigenetic dysregulation of imprinted genes in the chromosome 11p15.5 region. Primary diagnostic features are macroglossia, prenatal and postnatal overgrowth, and abdominal wall defects (1), with associated findings often including organomegaly, neonatal hypoglycemia, and structural cardiac anomalies (1). In this study, we present a case of BWS in a 2-month-old female infant, with genetically confirmed imprinting control region 1 (IC1) hypermethylation and reduced *H19* expression. This patient also presented with recurrent atrial tachycardia, a cardiac manifestation that is relatively uncommon in BWS.

## Case presentation

**Figure 1:**
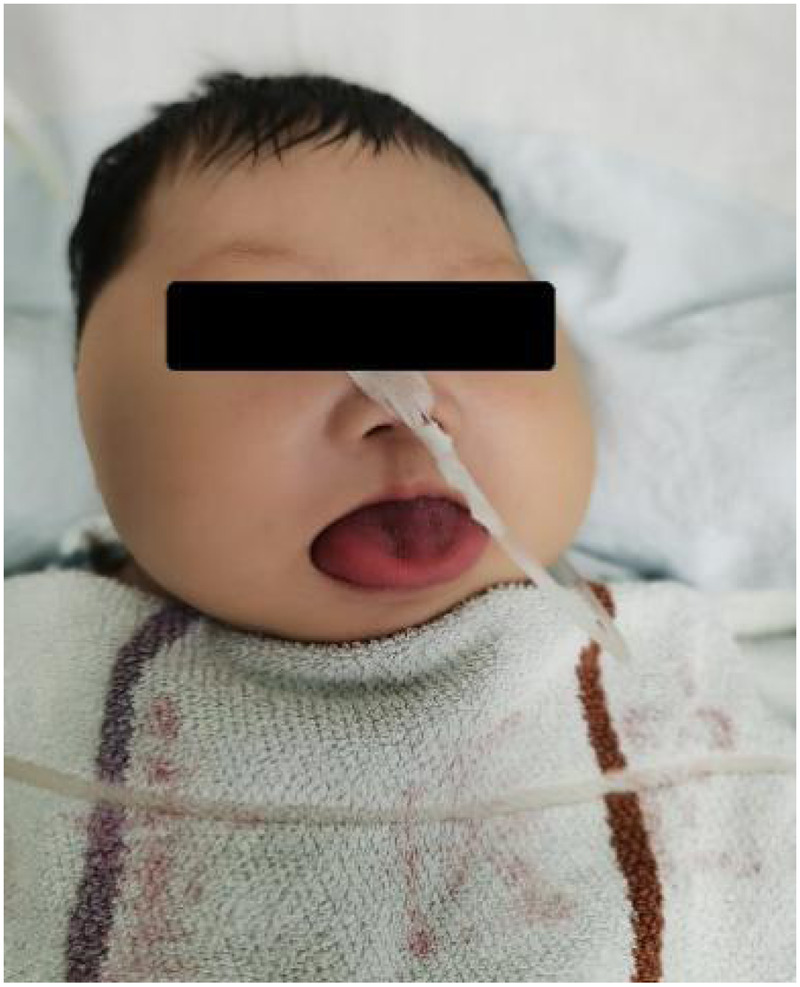
Clinical photograph showing macroglossia with the tongue protruding beyond the oral cavity at 2 months of age.

A two-month-old female infant presented with lethargy lasting 2 h and arrhythmia lasting 1.5 h. She had congenital macroglossia, with supine shortness of breath and apnea relieved by lateral positioning. A karyotype genetic analysis performed at another hospital showed a 46, XX karyotype. A brain magnetic resonance imaging (MRI) was performed, revealing no abnormalities in the brain parenchyma. Her tongue was enlarged. A physical examination on admission revealed a body weight of 7.9 kg and height of 61 cm. She was breathing steadily without cyanosis. She displayed no rash. Her facial appearance was symmetrical. She exhibited signs of macroglossia with protrusion beyond the oral cavity (Figure 1). No rales were auscultated in the lungs. Her heart sounds were clear, although her pulse was irregular with a heart rate of 184 beats/min. No murmurs were heard. She had a soft abdomen but a bulging umbilicus that could be retracted. Her liver was palpated 3 cm below the right costal margin and the spleen 2 cm below the left costal margin, both with a soft consistency and well-defined margins.

The patient was delivered at full term by cesarean section because of fetal macrosomia. Her mother had experienced two pregnancies with one living child (G2P1). The infant had a history of perinatal hypoxia and neonatal asphyxia. Antenatal polyhydramnios was detected at 28 weeks of gestation; however, data regarding the amount of amniotic fluid after delivery were unavailable. In the mother’s first pregnancy, an induced abortion at 8 weeks of pregnancy was performed because of the absence of fetal heart rate and fetal bud. The patient’s parents were healthy, with no family history of genetic disorders or congenital anomalies.

**Figure 2:**
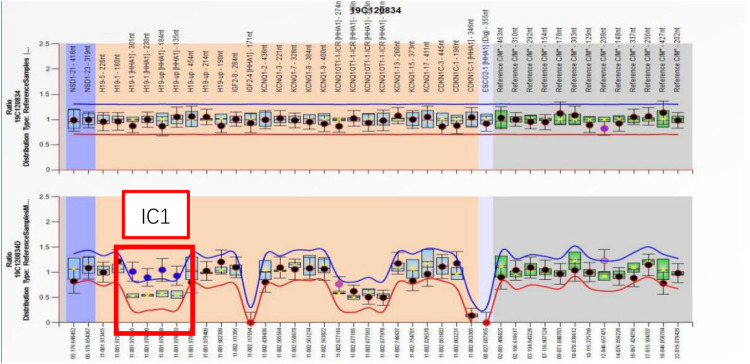
The MLPA assay reported a normal gene copy number on chromosome 11p15.5 but a gain of methylation at IC1.

A comprehensive set of investigations was conducted after admission. An electrocardiogram (ECG) revealed atrial tachycardia and paired or isolated premature ventricular contractions combined with aberrant ventricular conduction. An echocardiogram (Echo) revealed a patent ductus arteriosus measuring 1.7 mm, without myocardial hypertrophy. The dimensions of the cardiac chamber were normal. No anomalies were detected in the complete blood count (CBC), C-reactive protein (CRP), blood gas analysis, liver and kidney function testing, myocardial injury markers, blood sugar, thyroid function, and metabolic disease screening. A multiplex ligation–dependent probe amplification (MLPA) assay revealed a normal gene copy number on chromosome 11p15.5 but a gain of methylation at IC1 (Figure 2). A diagnosis of BWS was established.

**Figure 3:**
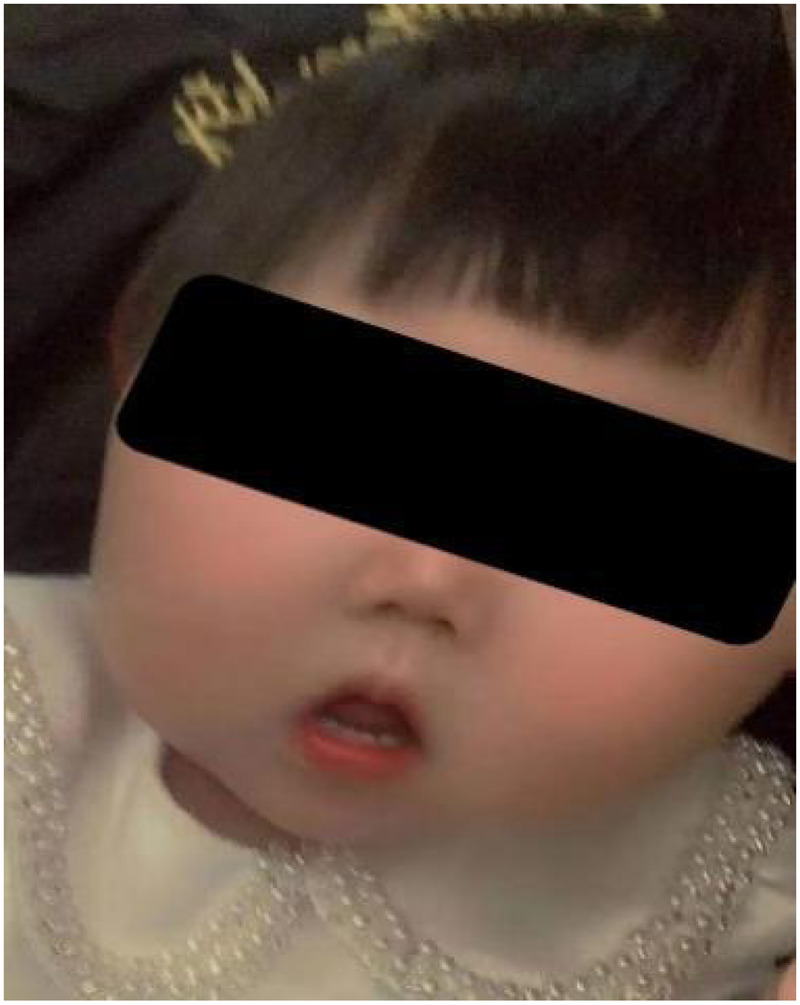
Postoperative photograph at 12 months showing reduced tongue volume following tongue reduction surgery and ablation therapy.

The patient’s atrial tachycardia was effectively controlled with metoprolol and digoxin on the third day. Repeat ECG and Holter monitoring showed normal results. No neoplastic lesions were found. Follow-up visits after discharge showed normal psychomotor development, with no reported hemihyperplasia. However, her enlarged tongue significantly impacted pronunciation and feeding, due to which a tongue reduction surgery combined with ablation therapy was performed at the age of 12 months (Figure 3). The patient’s speech improved postoperatively and she was able to pronounce two-syllable words by the age of 18 months. Regular follow-up examinations of abdominal and kidney ultrasonography and serum tumor biomarkers did not identify tumor lesions. Repeat echocardiography showed a patent ductus arteriosus measuring 1.2–1.7 mm, without myocardial hypertrophy. Atrial tachycardia was not detected by repeat ECG and Holter monitoring, and therefore, digoxin was withdrawn. However, atrial tachycardia recurred at 20 months of age, and this was managed by adjusting the dosage of metoprolol. This recurrence, despite the absence of myocardial hypertrophy, underscores the necessity of ongoing cardiac surveillance in BWS.

## Discussion

BWS is a congenital overgrowth disorder characterized by prenatal and postnatal macrosomia and an increased risk of embryonal tumors (1). It was first reported by Beckwith and Wiedemann in the 1960s, and has an estimated prevalence of 1:10,000–26,000 (2). Reports of BWS remain limited, and most cases occur in children, particularly among those conceived via assisted reproductive technologies (3). Although rare, adult presentations or diagnoses of BWS have also been documented (4).

## Molecular subtypes and pathogenesis of BWS

BWS is a genomic imprinting disorder. Imprinted genes are characterized by monoallelic expression in a parent-of-origin-specific manner; in other words, only the allele inherited from one parent (either maternal or paternal) is expressed, while the allele from the other parent is silenced. This monoallelic expression is primarily regulated by epigenetic mechanisms, including DNA methylation and histone modifications.

BWS is associated with varying alterations in the chromosome 11p15.5, leading to distinct phenotypes. Molecular aberrations can be detected in the majority of BWS patients (80%) (5–7). Specifically, gain of methylation at IC1 is found in 5%–10% of BWS patients. IC1 is a region of DNA that controls the expression of imprinted genes *H19* and *IGF2*. The *H19* gene is a maternally expressed allele and encodes untranslated RNA, serving as a tumor suppressor gene; on the other hand, the *IGF2* gene expresses the paternal allele and encodes a potent fetal growth factor. Gain of methylation at IC1 results in a biallelic expression of *IGF2* and silencing of *H19*. Conversely, loss of methylation at imprinting control region 2 (IC2), the most common molecular defect (approximately 50% of cases), results in a biallelic expression of the non-coding RNA *KCNQ1OT1* and downregulation of *CDKN1C*, a maternally expressed cell cycle inhibitor. A paternal uniparental disomy (UPD) of 11p15.5 accounts for approximately 20% of BWS cases, leading to a biallelic expression of *IGF2* and reduced expression of both *H19* and *CDKN1C*. Primarily sporadic mutations in *CDKN1C* are observed in approximately 5% of BWS cases. Paternal duplication or maternal translocation/inversion at 11p15.5 collectively accounts for another 1%. However, approximately 20% of BWS cases lack detectable molecular abnormalities, potentially because of tissue-limited mosaicism, uncharacterized epigenetic dysregulation, or novel pathogenic mechanisms. Recent studies have further highlighted the complexity of the BWS pathogenesis, demonstrating that multilocus imprinting disturbances and tissue-specific mosaic epigenetic alterations may contribute to phenotypic heterogeneity (8–10). In addition, advances in methylation profiling and high-throughput sequencing have offered new insights into genotype–phenotype correlations and refined surveillance strategies.

## Phenotypic heterogeneity and diagnostic criteria in BWS

The clinical manifestations of BWS in infants and children vary with molecular defects and tissue mosaicism on chromosome 11p15.5. The diagnostic criteria for BWS have evolved over time. The European Cooperation in Science and Technology (COST) established a collaborative network for BWS research and management (1). This network developed consensus guidelines for the diagnosis and treatment of BWS, proposing that BWS can range from a classic presentation to an isolated lateralized overgrowth (hypertrophy) (9). A scoring system is used for diagnosis. Each dominant clinical manifestation—macroglossia, omphalocele, lateralized overgrowth, bilateral Wilms’ tumor, hyperinsulinism, and pathological findings of adrenal cortical hyperplasia, placental mesenchymal dysplasia, or pancreatic adenomatous disease—is assigned 2 points. Supportive features—such as birthweight greater than 2 SD above the mean, nevus simplex, polyhydramnios, ear creases, transient neonatal hypoglycemia, BWS-associated tumors, organomegaly, or umbilical defects—are each assigned 1 point. A clinical score greater than or equal to 4 points is sufficient to establish a diagnosis of classic BWS, even without molecular confirmation. Genetic testing is recommended for individuals scoring greater than or equal to 2 points. Those scoring less than 2 are not candidates for testing, while individuals with greater than or equal to 2 points but negative genetic findings require further evaluation or referral to BWS experts. The present patient scored more than 4 points based on cardinal and supportive features. Combined with the MLPA finding, she was finally diagnosed with BWS.

Congenital heart disease is the most common cardiac anomaly in patients with BWS (11). Previous studies have reported that patients with BWS may present with long QT syndrome (LQTS), which has been associated with copy number variations or genomic rearrangements involving the IC2 region (12, 13). Non-inflammatory localized myocardial hypertrophy and secondary supraventricular tachycardia are also occasionally reported in patients with BWS (14, 15). The patient with BWS in this study was diagnosed with a patent ductus arteriosus and atrial tachycardia. Despite the absence of myocardial hypertrophy, cardiac follow-up is warranted given the potential for arrhythmias associated with structural progression in BWS. In this context, the recurrent atrial tachycardia observed in our patient may represent a primary arrhythmia rather than a secondary manifestation of hypertrophic changes, underscoring the need for further investigation into the arrhythmogenic mechanisms of BWS.

## Genotype–phenotype correlation of BWS

Gain of methylation at IC1 is associated with an increased risk of macroglossia, umbilical hernia, and nephroblastoma (16), while loss of methylation at IC2 is more frequently associated with omphalocele, macroglossia, ear creases and/or pits, facial nevus simplex, and prematurity, but confers a relatively low risk of tumor development (17). A paternal UPD of 11p15.5 carries a high risk of tumor development, mainly hepatoblastoma and nephroblastoma (1). Paternal UPD mosaicism is strongly associated with lateralized overgrowth (10). Polyhydramnios is reported in some BWS cases with hypermethylation at IC1 (11). In the present case, the patient—with a gain of methylation at IC1—had typical features of BWS, such as polyhydramnios, macrosomia, and umbilical hernia. A reduced *H19* expression was detected, warranting long-term follow-up to monitor tumor risk. Currently, there is no established correlation between IC1 hypermethylation and atrial tachycardia in the literature; therefore, this finding in our patient should be interpreted with caution, and additional studies should be conducted to clarify whether such an association exists.

Reduced expression of the H19 gene, a maternally expressed long non-coding RNA with tumor-suppressive properties, may have important implications in Beckwith–Wiedemann syndrome. H19 normally antagonizes IGF2 activity and regulates cell proliferation and differentiation through multiple pathways, including modulation of microRNAs and epigenetic regulators. Its downregulation has been associated with enhanced IGF2 signaling, increased cell growth, and tumor susceptibility. Although gain of methylation at IC1 is a well-established cause of H19 silencing, the specific contribution of reduced H19 expression to the phenotype of BWS, particularly in relation to arrhythmogenesis, remains unclear. Further studies are warranted to elucidate the mechanistic role of H19 loss in the clinical heterogeneity of BWS.

## Treatment and follow-up

The prognosis of BWS depends primarily on neonatal complications and tumors (11). Life-threatening complications in the neonatal period are hypoglycemia, macroglossia-induced respiratory obstruction, and giant omphaloceles. While the risk of tumor development in BWS is highest during infancy and early childhood, it does not increase significantly in adolescence or adulthood; however, late-onset complications such as renal medullary dysplasia and male subfertility have been reported and warrant clinical attention (18). Therefore, treatment and follow-up strategies for BWS should be guided by the patient's specific complications and molecular subtype.

Surgery is required for infants with BWS who have macroglossia that results in breathing problems, obstructive sleep apnea, feeding difficulties, persistent drooling, and speech and joint issues. During the neonatal period, airway compromise is the primary surgical indication. More favorable outcomes are generally achieved when surgery is performed prior to 2–3 years of age (11, 19). The patient in this study exhibited shortness of breath and breath-holding in the supine position after birth. However, surgical intervention was deferred as her symptoms were relieved by lateral positioning. At 12 months of age, because of persistent feeding difficulties and delayed speech development, tongue reduction surgery with ablation therapy was performed.

The risk of embryonal tumors in children with BWS is estimated to be approximately 8%, and both tumor type and incidence vary based on molecular subtype (9, 20). Tumor screening and monitoring are essential for children diagnosed with or suspected of having BWS (21). The American Association for Cancer Research (AACR) recommends abdominal ultrasound every 3 months until the age of 4 years, and renal and adrenal ultrasound every 3 months until the age of 7 years. Serum alpha-fetoprotein (AFP) levels should also be measured every 3 months until the age of 4. COST suggests an abdominal ultrasound at an interval of 3 months until the age of 7. Our follow-up strategy, which includes an abdominal ultrasound and serum AFP measurement every 3 months, is consistent with the AACR recommendations for IC1 hypermethylation, while also aligning with COST guidelines for tumor surveillance in BWS. Given the presence of IC1 hypermethylation, our patient was at an elevated risk for Wilms’ tumor and required careful tumor surveillance. In addition, owing to the presence of both patent ductus arteriosus and atrial tachycardia, echocardiographic follow-up was scheduled every 3 months to evaluate cardiac morphology and detect potential progression to myocardial hypertrophy. At 20 months, follow-up echocardiography confirmed a persistent patent ductus arteriosus without evidence of myocardial hypertrophy. However, the recurrence of atrial tachycardia at 20 months underscores the importance of long-term cardiac monitoring, given the potential for progression to structural changes such as non-inflammatory myocardial hypertrophy.

Because of the need for multidisciplinary follow-up visits every 3 months—involving the cardiology, dentistry, and oncology departments across multiple hospitals—the patient's family faced a substantial financial burden. In addition, ongoing anxiety about the risk of tumor caused psychological distress, adversely impacting the family's work and overall quality of life.

Although rare in occurrence, BWS can have diverse clinical phenotypes. BWS affects multiple systems and organs and, notably, also increases the risk of embryonal tumors during early childhood. Multidisciplinary collaboration is crucial for diagnosing and managing BWS, while long-term follow-up is vital for monitoring complications and tumors to improve outcomes. Support for families of children with BWS may help improve adherence to follow-up and contribute to more effective long-term management of the condition.

## Data Availability

The data used to support the findings of this study are available from the corresponding author upon request.
